# A Remarkable Suicide

**Published:** 1890-08

**Authors:** F. S. White

**Affiliations:** Assistant Superintendent State Lunatic Asylum, Terrell, Texas


### A REMARKABLE SUICIDE.

BY DR. F. S. WHITE, ASSISTANT SUPERINTENDENT STATE LUNATIC ASYLUM, TERRELL, TEXAS.

[Paper read before the Terrell Medical Society, and voted for publication in DANIEL'S TEXAS MEDICAL JOURNAL.]

J. W., male, age thirty-five, married, had been insane some seven months, and confined in the Asylum two months. His insanity was of a melancholic nature. He had the delusion that some one wanted to kill him, and that everybody was his enemy. Two weeks ago he had so far recovered that preparations were being made to send him home, but the excitement incident to going home overbalanced his mind again, and the same delusions returned and were now more intense.

On May 16th he was outside in the grounds with the other patients, when a sudden impulse to destroy himself seized him, and without any warning he ran, head foremost, against a tree from a distance of about twenty feet. He fell perfectly limp and lifeless to the ground. I saw him in about five minutes; found him wholly unconscious, pulse slow, about forty-five, respiration slowed, pupils contracted, could not swallow. Had not, up to this time, moved a single muscle since the injury.

I had him placed on a stretcher, carried to the house and placed in bed. In half an hour I again examined him; found some reaction, pulse now about fifty, and respiration fifteen; pupils not so closely contracted, some motion in upper extremities, and he could now swallow. No motion or sensation from about the sixth rib downward. Very little sensation in forearm; could not talk or answer questions intelligently. Breathing was abdominal; sensation normal in face and neck; he could move his head but complained of pain in his neck. I did not examine his neck very closely but examined the head at seat of injury and found no indication of fracture whatever. He remained unchanged for twelve hours, when there was considerable reaction; pulse increased to sixty and respiration to sixteen, pupils nearer normal but still contracted. He could be turned in bed and seemed to rest better on his side, still, while being moved he complained of pain in his neck and back, also said he had headache. It was now found that his bladder and bowels were paralyzed; could not void his urine, and it was with great difficulty that his bowels could be made to move. The bladder was emptied with a catheter. There was no change in his condition for two days, when there was a very slight rise of temperature and his bowels acted several times. He continued in practically the same condition until the 20th, four days after the injury, when the symptoms became more grave. There were contractions of the muscles of the face and arms; breathing more rapid and pulse weaker and weaker until he died.

I proceeded to make an autopsy some eighteen hours after death. The scalp was only slightfy bruised at the seat of the injury and was normal in other respects. Skull of normal thickness and appearance. No indication on either external or internal table of any injury. The dura mater was considerably congested and adhered to the pia mater, along the course of the superior longitudinal sinus, but this was chronic and not in any way affected by the recent injury. In all other localities the dura mater was normal, with the exception of the congestion. The pia mater was considerably congested throughout, especially so at the base of the brain. There was a small blood clot just beneath the tentorium cerebelli and resting on the cerebellum. An enormous quantity of blood escaped from the sinuses. The brain was considerably congested, the puncta being well marked throughout. Normal quantity of fluid in the ventricles.

I now turned the body upon its face preparatory to examining the spinal cord. I made an incision from the first cervical vertebræ to the last lumbar, directly down to the spinous processes; disected the muscles nicely down to the laminæ on each side, and with a carpenter's joining saw I cut through the laminæ, and with a strong costatome raised off the spinous processes and exposed the cord.

I found a fracture of the fifth and sixth cervical vertebræ. The spinous process of the fifth was fractured and the laminæ and body of the sixth, the body being fractured vertically and through the centre. The fragments of the laminæ of the fifth were pressing on the cord whose membranes were intensely congested. There was also at this point a small blood clot. The central part of this portion of the cord was found to be broken down to such an extent that it would not hold together. This condition involved about two inches of the chord. The entire cord was congested, but at the third dorsal vertebræ was found another small blood clot, and the congestion around this was more marked than at other points. There were also at a few points small inflammatory patches.

The nature and extent of this injury to the cord was such that no motor or sensory impressions could pass it; still there were nervous action and force manifested below the seat of the lesion. Now there was total paralysis of both motion and sensation in the lower extremities and partial paralysis of the upper; also some considerable impairment of the respiratory functions. The pneumogastric nerve, before its exit through the jugular foramen receives filaments from the spinal cord as low down as the sixth cervical nerve. The injury to this portion of its origin was the cause of the interference with respiration. The phrenic nerve which aids in respiration, is given off from the fourth and fifth cervical. The brachial plexus is formed from the fourth, fifth, sixth, seventh and eighth cervical and the first dorsal nerves. Now, that portion which originated in the fourth cervical was uninjured, and hence we account for the partial motion in the upper extremities.

